# Histone lactylation: a metabolic-epigenetic bridge linking glycolysis to salivary gland fibrosis in Sjögren’s disease

**DOI:** 10.3389/fimmu.2026.1917176

**Published:** 2026-09-22

**Authors:** Yang Qin, Huayan Tang

**Affiliations:** Shanghai Municipal Hospital of Traditional Chinese Medicine, Shanghai University of Traditional Chinese Medicine, Shanghai, China

**Keywords:** epigenetic regulation, histone lactylation, lactate microenvironment, metabolic reprogramming, salivary gland fibrosis, Sjögren’s disease

## Abstract

Sjögren’s disease (SjD) is a chronic, systemic autoimmune disorder characterized by lymphocytic infiltration and progressive loss of exocrine gland function. Despite advances in understanding its immunopathogenesis, the molecular mechanisms governing the transition from inflammation to irreversible salivary gland fibrosis remain obscure. Emerging evidence suggests that the crosstalk between metabolic reprogramming and epigenetic modifications profoundly shapes the local inflammatory microenvironment and accelerates disease progression. Histone lactylation, a recently discovered lactate-derived epigenetic mark, directly couples enhanced glycolytic activity and lactate accumulation with transcriptional regulation, thereby serving as a pivotal metabolic-epigenetic bridge. This review systematically examines the potential role of histone lactylation in SjD salivary gland pathology. We delineate the enzymatic machinery of lactylation, the metabolic origins of lactate within the inflamed microenvironment, and the functional implications for immune cell polarization, epithelial-mesenchymal transition, and myofibroblast activation. While direct evidence in SjD is currently lacking, we synthesize findings by integrating immunometabolism, epigenetic rewiring, and tissue fibrogenesis, we aim to establish histone lactylation as a transformative framework for understanding SjD pathogenesis and highlight its translational potential for novel biomarkers and therapeutic interventions.

## 1 Introduction

Sjögren’s disease (SjD) is a chronic, systemic autoimmune disease that primarily affects the salivary and lacrimal glands, leading to debilitating dry mouth and dry eye symptoms (1). Beyond glandular manifestations, SjD can involve systemic complications, including pulmonary interstitial disease, renal tubular acidosis, peripheral neuropathy, and an increased risk of lymphoproliferative disorders, substantially impairing patients’ quality of life and increasing mortality (2). Epidemiological studies indicate that SjD affects approximately 0.1% to 0.6% of the population, predominantly middle-aged women, with a female-to-male ratio of about 9:1 (3). Despite growing insights into disease mechanisms, the fundamental causes of persistent inflammation and irreversible glandular damage remain incompletely understood, and disease-modifying therapies are limited. Progressive fibrosis of the salivary glands is a major contributor to the permanent loss of secretory function, yet how metabolic disturbances drive this fibrotic process through epigenetic reprogramming is not well defined (4).

Epigenetic modifications—heritable changes in gene expression without alterations to the DNA sequence—have emerged as critical regulators of immune cell function and tissue homeostasis in autoimmune diseases (5). Among these, histone post-translational modifications (PTMs) play a central role in chromatin remodeling and transcriptional regulation. Classical histone acetylation, methylation, and phosphorylation have recently been joined by novel acylations, including crotonylation, beta-hydroxybutyrylation, and lactylation (6). In the context of SjD, multiple epigenetic mechanisms have been implicated. DNA methylation abnormalities are well-documented, with global hypomethylation in peripheral blood mononuclear cells and disease-specific hypermethylation at promoters of interferon signaling and B cell regulatory genes (7, 8). Histone acetylation and methylation marks, particularly at H3 and H4 residues, regulate the expression of key inflammatory genes in SjD salivary glands and lymphocytes (9). MicroRNAs (miRNAs), including miR-146a, miR-155, and the miR-17–92 cluster, are dysregulated in SjD and modulate pathways from type I interferon production to B cell hyperactivation (10, 11). Chromatin remodeling and non-coding RNA networks further contribute to the epigenetic landscape of SjD (12). Histone acetylation, in particular, has been shown to regulate the immune microenvironment by promoting the transcription of pro-inflammatory cytokines (e.g., TNF-α, IL-6) and chemokines (e.g., CXCL10, CCL5) in salivary gland epithelial cells (SGECs) and infiltrating lymphocytes, thereby sustaining local inflammation (9, 13).

Recent studies have revealed that enhanced glycolysis, mitochondrial dysfunction, and increased lactate production are metabolic hallmarks of both immune cells and SGECs in SjD patients (14–16). The SjD salivary gland microenvironment—characterized by chronic inflammation, tissue hypoxia, and dysregulated immune cell activation—creates conditions favorable for aerobic glycolysis (the Warburg effect), resulting in elevated lactate levels (17). Metabolomic studies have confirmed elevated lactate in the saliva of SjD patients, reflecting enhanced glycolytic activity in affected glands (18). Given that histone lactylation is highly sensitive to intracellular lactate concentrations, these metabolic perturbations may have far-reaching epigenetic consequences (6). Mitochondrial dysfunction further exacerbates this metabolic shift by impairing oxidative phosphorylation and promoting a reliance on glycolysis for ATP production, thereby increasing lactate generation (16). This establishes a metabolite (lactate) → modification (lactylation) → phenotype (inflammation/fibrosis) axis that may be central to SjD pathogenesis.

Histone lactylation, first reported in 2019, serves as a direct molecular bridge connecting cellular metabolic states to gene transcriptional regulation (6). Although no studies have yet directly demonstrated histone lactylation’s involvement in SjD pathogenesis, emerging evidence from tumor, fibrosis, and inflammatory disease models indicates that this modification regulates macrophage polarization, lymphocyte differentiation, cytokine production, and tissue remodeling. The purpose of this review is to synthesize evidence from these related fields and construct a testable framework for the role of histone lactylation in SjD. We will systematically examine the molecular mechanisms of histone lactylation, the metabolic landscape of SjD salivary glands, its functional consequences on immune cells and fibrogenesis, and the therapeutic potential of targeting this axis. By integrating immunometabolism, epigenetics, and tissue fibrosis, we propose a model wherein lactate-driven histone lactylation promotes the progression from inflammatory damage to irreversible glandular fibrosis in SjD, identifying promising therapeutic strategies to interrupt this pathogenic cascade.

## 2 Metabolic reprogramming and the lactate microenvironment in Sjögren’s disease

Immune cell activation and differentiation depend on adaptive metabolic pathway adjustments (19). Quiescent T cells and regulatory T cells (Tregs) primarily rely on oxidative phosphorylation (OXPHOS) and fatty acid oxidation, whereas activated effector T cells, M1 macrophages, and B cells undergo a metabolic switch toward aerobic glycolysis to meet the demands of rapid proliferation and effector molecule synthesis (20, 21). This metabolic plasticity becomes pathologically entrenched in autoimmune diseases, where pro-inflammatory cells maintain a hyper-glycolytic state, providing a foundation for subsequent epigenetic reprogramming (22).

In SjD, metabolic reprogramming has been demonstrated across multiple cell types (14). In CD4+ T cells, overactivation of the mTOR-HIF-1α pathway drives glycolytic gene expression, promoting Th1/Th17 differentiation while undermining Treg stability (23). B cells exhibit upregulated glucose uptake and mitochondrial OXPHOS due to enhanced BAFF signaling and sustained PI3K-AKT-mTOR pathway activation (24). SGECs show significantly upregulated LDHA and PKM2 expression under IFN-γ/TNF-α stimulation, accompanied by decreased mitochondrial membrane potential, indicating functional impairment (16). Metabolomic studies have confirmed elevated lactate levels in the saliva of SjD patients (18). While direct measurement of lactate concentrations within salivary gland tissue is limited, the consistent elevation of salivary lactate, together with upregulated expression of glycolytic enzymes in SjD SGECs and infiltrating immune cells, strongly suggests a lactate-enriched local microenvironment (25).

Mitochondrial dysfunction plays a critical role in this metabolic shift. Mechanistically, mitochondrial dysfunction in SjD SGECs and infiltrating immune cells manifests as decreased mitochondrial membrane potential, impaired electron transport chain activity, and elevated reactive oxygen species (ROS) generation. This mitochondrial impairment creates a vicious cycle: ROS stabilize hypoxia-inducible factor-1α (HIF-1α) by inhibiting prolyl hydroxylase activity, while concomitant ATP deficiency activates AMPK, which in turn synergizes with HIF-1α to drive the transcription of glycolytic enzymes including HK2, PKM2, and LDHA. The resulting glycolytic dependency further increases lactate production, exacerbating mitochondrial dysfunction through lactate-mediated inhibition of mitochondrial complex activity. This mitochondrial-glycolytic feedback loop establishes a self-sustaining metabolic circuit that not only fuels energy production through aerobic glycolysis but also provides the substrate—lactate—for subsequent histone lactylation. Notably, mitochondria-related genes are differentially expressed in pSS salivary glands, and innate immune cells such as monocytes and M1 macrophages exhibit a predominant reliance on glycolysis rather than oxidative phosphorylation, highlighting the cell-type-specific nature of this metabolic reprogramming.In SjD, SGECs and immune cells exhibit impaired mitochondrial respiration, reduced ATP production, and increased reactive oxygen species (ROS) generation (16). This dysfunction not only compromises energy production but also stabilizes HIF-1α, which in turn drives the transcription of glycolytic enzymes, creating a vicious cycle of mitochondrial impairment and glycolytic dependency (26). Furthermore, macrophage metabolic reprogramming is pivotal in salivary gland fibrosis. M1 macrophages, which predominate in SjD salivary glands, rely on glycolysis and produce pro-inflammatory cytokines that sustain inflammation (27). This glycolytic metabolism generates lactate, which can act in a paracrine manner to activate neighboring fibroblasts (28). A positive feedback loop exists between type I interferon signaling and glycolytic reprogramming (29). IFN-α/β activates STAT1 via the JAK-STAT pathway, which binds to the HIF1A promoter, upregulating HIF-1α and inducing glycolytic enzymes including HK2, PKM2, and LDHA (30). Thus, sustained activation of the IFN-JAK-STAT-HIF-1α-LDHA-lactate pathway converts inflammatory signals into metabolic outputs, providing lactate for histone lactylation. We propose this as a working model—a metabolic-epigenetic feedback loop—wherein inflammation drives glycolysis, glycolysis generates lactate, and lactate, through histone lactylation, reinforces the transcription of inflammatory and fibrotic genes. This model, while conceptually compelling, requires systematic experimental validation in SjD-specific systems.

## 3 Molecular basis of histone lactylation and its adaptation to the SjD microenvironment

Histone lactylation is a novel epigenetic modification derived from lactate that directly reflects cellular glycolytic status (6, 31). Its molecular regulatory system comprises three classes of functional enzymes:

Writers: p300/CBP was the first identified lactyltransferase, catalyzing the transfer of lactyl-CoA to histone lysine residues (32). GCN5 (KAT2A) mediates H3K18la in M2 macrophages, HBO1 (KAT7) preferentially targets H3K9la, and KAT8 (MOF) also exhibits non-histone lactylation activity (33, 34). The dual acetylation/lactylation activity of p300/CBP suggests that the relative abundance of lactyl-CoA versus acetyl-CoA determines modification type preference—under high lactate conditions, lactylation may competitively displace acetylation, reshaping the epigenetic landscape (32).

Erasers: Class I HDACs (HDAC1-3) and SIRT1–3 possess delactylase activity, with SIRT2 showing the highest efficiency for lactyl group removal (35–37). This implies that HDAC/SIRT inhibitor use may inadvertently upregulate lactylation levels, necessitating careful evaluation in therapeutic strategies.

Readers: TRIM33 was the first identified lactylation-specific reader, with its bromodomain showing higher affinity for lactylated lysine than for acetylated lysine (38). BRG1 recognizes H3K18la and activates transcription through chromatin remodeling (39), while DPF2 recognizes H3K14la (40).

Over twenty histone lactylation sites have been identified, with H3K18la being the most extensively studied (41). ChIP-seq analyses demonstrate that H3K18la enriches at transcription start sites and active enhancer regions, where it weakens histone-DNA interactions, enhancing transcription factor accessibility. In M1 macrophages, LPS-induced glycolysis produces lactate that places H3K18la marks at M2 marker gene (Arg1, Vegfa, Klf4) promoters, preparing the transcriptional groundwork for transition to the M2 phenotype during inflammation resolution—a metabolic memory mechanism with important implications for macrophage dynamics in SjD (42–44).

In the context of SjD, the lactate-enriched microenvironment of the salivary gland is expected to drive analogous histone lactylation events in infiltrating immune cells and resident stromal cells. For T cells, Th17 differentiation depends on glycolysis, and histone lactylation at the IL17A and RORC loci promotes pathogenic effector function (45). Treg cells, which rely primarily on OXPHOS, maintain low baseline lactylation, but inflammation-induced lactylation can modulate Foxp3 expression (46). In B cells, the high glycolytic rate of germinal center B cells correlates with lactylation at immunoglobulin and class-switch region genes, potentially regulating antibody production (47). These findings suggest that histone lactylation may contribute to the Th17/Treg imbalance and B cell hyperactivation characteristic of SjD, though direct evidence is lacking.

## 4 Lactylation-mediated regulation of immune cell function

Given the central role of immune dysregulation in SjD, understanding how histone lactylation modulates immune cell function is critical. This section focuses on T cells, B cells, and macrophages, as they are key players in SjD pathogenesis.

Macrophages: Macrophages represent the most extensively studied model. M1 polarization is accompanied by enhanced glycolysis and lactate production, yet lactate accumulation establishes H3K18la enrichment at M2 gene promoters, pre-opening chromatin for phenotype transition (42). This mechanism acts as a built-in timer—after inflammatory signals diminish, the prepared chromatin state enables rapid M2 gene activation, promoting tissue repair (43). In SjD, where M1/M2 imbalance in salivary glands has been established, lactylation may serve as a molecular switch regulating this balance (27). Importantly, M1 macrophages secrete pro-fibrotic factors such as TGF-β, which can induce fibroblast-to-myofibroblast transformation, directly linking macrophage lactylation to glandular fibrosis (48).

T Cells: Th17 differentiation depends on glycolysis, with increased histone lactylation at IL17A and RORC loci promoting pathogenic effector function (45). Treg cells, relying primarily on OXPHOS, maintain low baseline lactylation, but inflammation-induced lactylation can modulate Foxp3 expression (46). CD8+ T cell effector genes (perforin, granzyme B, IFN-γ) show H3K18la and H3K9la enrichment at their promoters that positively correlates with cytotoxic function (49). The Th17/Treg imbalance is a recognized immunological feature of SjD, and lactylation’s contribution to this imbalance warrants investigation.

B Cells: The high glycolytic rate of germinal center B cells correlates with lactylation at immunoglobulin loci and class-switch region genes, potentially regulating antibody class switching and affinity maturation (47). SjD is characterized by heightened B cell activation and autoantibody production (anti-Ro/SSA and anti-La/SSB) (50). Whether lactylation participates in abnormal autoantibody production remains unknown, but represents an important area for future research. Additionally, tertiary lymphoid structures (TLS), which are organized aggregates of B cells, T cells, and stromal cells, form in SjD salivary glands and support local autoantibody production (51). The metabolic environment within TLS may promote histone lactylation in germinal center B cells, potentially sustaining local autoreactive responses.

## 5 Crosstalk between histone lactylation and major signaling pathways in Sjögren’s disease

Histone lactylation does not operate in isolation but intersects with multiple signaling pathways central to SjD pathogenesis. Importantly, this crosstalk is bidirectional: lactylation modulates pathway activity, and pathway activation, in turn, influences glycolysis and lactate production, creating positive feedback loops that sustain chronic pathology.

### 5.1 JAK/STAT

In SjD, the type I interferon signature is driven by the JAK-STAT pathway. Emerging evidence from other disease models suggests that histone lactylation may potentiate JAK-STAT signaling. For example, in models of arterial calcification, H3K18la has been shown to regulate the expression of CHI3L1, which in turn modulates inflammatory signaling (52). Conversely, IFN-α/β-activated STAT1 directly binds to the HIF1A promoter, upregulating glycolysis and lactate production (30). This creates a feedforward loop wherein JAK-STAT activation enhances lactate availability, promoting histone lactylation, which may further amplify STAT-dependent transcription of interferon-stimulated genes (ISGs). Whether H3K18la directly regulates STAT1-dependent ISG transcription in SjD salivary glands remains to be investigated.

### 5.2 NF-κB

NF-κB signaling, a central driver of salivary gland inflammation, can be directly activated by histone lactylation. In renal fibrosis models, lactate drives H3K18la at the Rela promoter, activating NF-κB and downstream inflammatory cascades (53). This mechanism may be particularly relevant to SjD, where SGECs exhibit constitutive NF-κB activation and produce chemokines (CXCL10, CCL5) that sustain local inflammation. In macrophages, NF-κB activation promotes NLRP3 inflammasome activation and IL-1β release, further amplifying inflammation (54). Conversely, TNF-α-mediated NF-κB signaling in SGECs upregulates LDHA expression, increasing lactate production and potentially enhancing lactylation in an autocrine manner (16).

### 5.3 mTOR/PI3K/Akt

The mTOR-HIF-1α axis is a well-established driver of glycolytic reprogramming in SjD T cells (23). Histone lactylation may reinforce this pathway through feedforward mechanisms: HIF-1α-driven glycolysis generates lactate, which in turn promotes H4K12la-dependent PIK3R2 expression, further activating PI3K/Akt/mTOR signaling (55). This positive feedback loop could explain the persistence of glycolytic metabolism in SjD-infiltrating lymphocytes.

### 5.4 TGF-β/SMAD

TGF-β/SMAD signaling is the principal driver of salivary gland fibrosis and EMT in SjD. In renal and pulmonary fibrosis models, lactate-induced histone lactylation directly upregulates TGF-β1 expression, which in turn activates Smad3 to drive fibrogenic gene transcription (56). This lactylation-TGF-β-Smad axis may constitute a self-amplifying loop in SjD salivary glands, where TGF-β produced by inflamed epithelia and infiltrating immune cells further promotes glycolysis and lactate production in neighboring fibroblasts, creating a pro-fibrotic microenvironment (57).

### 5.5 NLRP3

NLRP3 inflammasome activation has been implicated in SjD pathogenesis, particularly in SGEC pyroptosis. In other inflammatory models, glycolysis-dependent histone lactylation via the ROS/HIF-1α pathway activates NLRP3 and downstream IL-1β release (54). Whether H3K18la-driven NLRP3 activation contributes to SGEC damage in SjD warrants investigation.

Collectively, these findings suggest that histone lactylation serves as a metabolic-epigenetic node that integrates and amplifies signals from JAK/STAT, NF-κB, mTOR, TGF-β/SMAD, and NLRP3 pathways, creating a networked inflammatory circuitry that sustains chronic salivary gland pathology. The cell-type specificity of these interactions is crucial: NF-κB drives chemokine release in SGECs but inflammasome activation in macrophages; TGF-β induces EMT in epithelial cells and collagen deposition in fibroblasts.

## 6 Role of histone lactylation in Sjögren’s disease salivary gland pathology

The convergence of metabolic dysregulation, chronic inflammation, and epigenetic remodeling in SjD salivary glands provides compelling rationale for investigating histone lactylation in disease pathogenesis. Multiple lines of indirect evidence support the hypothesis that elevated lactate production drives pathological histone lactylation, leading to immune dysregulation, epithelial dysfunction, and progressive fibrosis (**Figure 1**).

**Figure 1:**
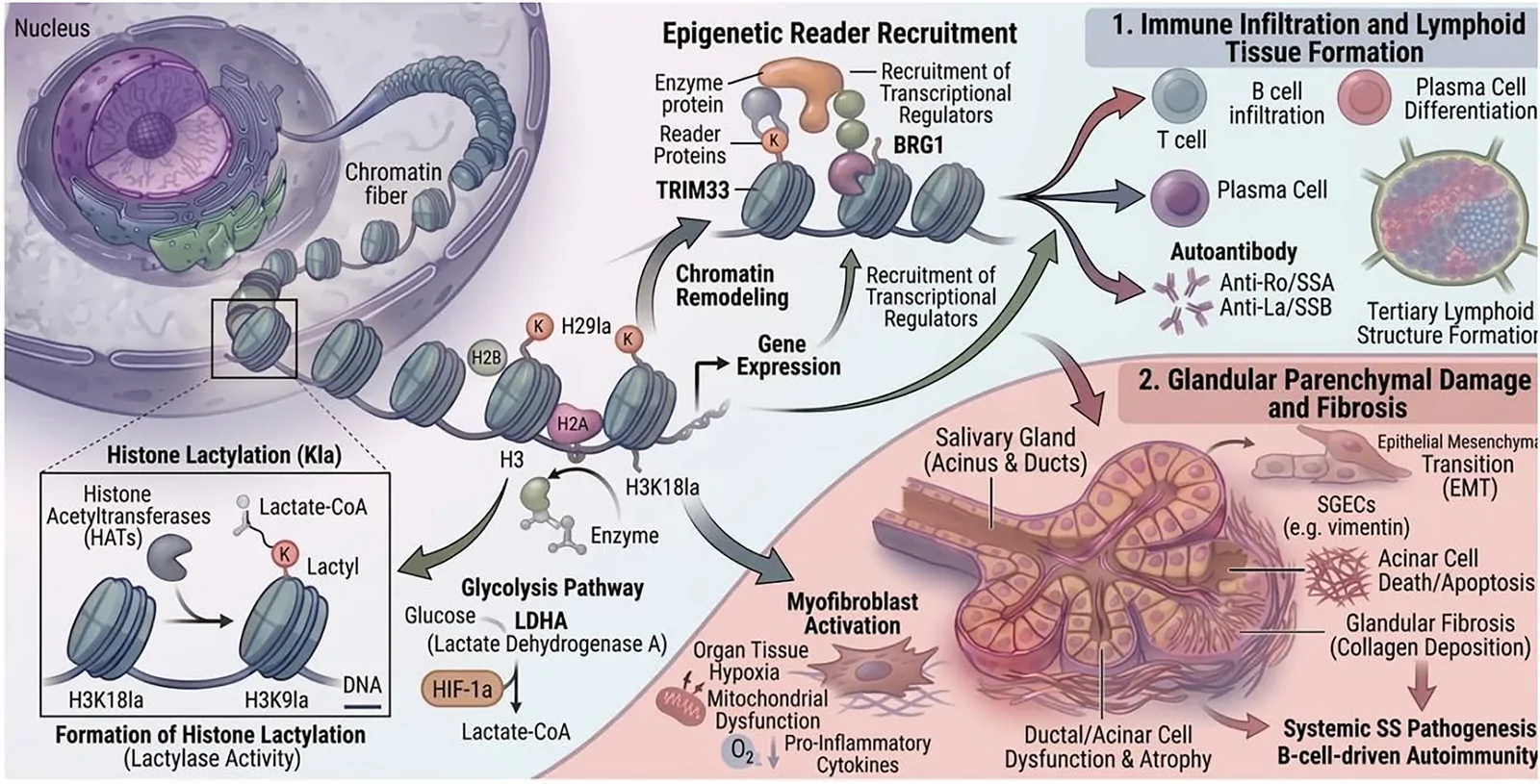
Proposed model of histone lactylation in sjögren’s disease pathogenesis. This schematic illustrates the hypothesized mechanisms linking histone lactylation to SjD. Bottom Left: Metabolic alterations in the inflamed salivary gland microenvironment—driven by glycolysis in immune cells (T cells, B cells, macrophages) and SGECs, mitochondrial dysfunction, and tissue hypoxia—lead to elevated intracellular lactate. Lactate is converted to Lactyl-CoA, which serves as a substrate for histone acetyltransferases (HATs, e.g., p300), resulting in lactylation of specific histone lysine residues (e.g., H3K18la, H3K9la). Middle: These lactylation marks are recognized by reader proteins (e.g., TRIM33, BRG1), promoting an open chromatin state and transcriptional activation. This drives the expression of pro-inflammatory cytokines (TNF-α, IL-6, IL-1β), type I interferon pathway genes (IRF7, ISG15), and B cell activation factors (BLIMP-1, BAFF). Right: Enhanced cytokine production and immune cell infiltration lead to salivary gland pathology: immune cell infiltration and lymphoid organization; ductal and acinar cell dysfunction and atrophy, causing hyposalivation; and glandular fibrosis driven by fibroblast activation and ECM deposition. Activated B cells and plasma cells produce anti-Ro/SSA and anti-La/SSB autoantibodies. Overall, histone lactylation serves as a key metabolic-epigenetic bridge integrating glycolytic metabolism with transcriptional reprogramming to drive SjD-associated inflammation, autoimmunity, and fibrosis. This model, while supported by evidence from other diseases, requires direct experimental validation in SjD. SGEC, salivary gland epithelial cell; ECM, extracellular matrix.

### 6.1 Lactate accumulation in the SjD salivary gland microenvironment

Targeted metabolomics studies have consistently reported significantly elevated lactate levels in the saliva of SjD patients (18). Immunohistochemical studies have demonstrated upregulated LDHA expression in acinar epithelial cells within inflammatory infiltrates, correlating positively with local inflammation scores (14). These findings suggest, though do not directly prove, that the tissue microenvironment is lactate-rich. The sources of this elevation are multi-cellular: activated T/B cells and M1 macrophages utilize aerobic glycolysis; SGECs upregulate LDHA and PKM2 under IFN-γ and TNF-α stimulation (16); and HIF-1α expression is elevated in SjD salivary glands, driving glycolytic enzyme transcription (23). However, it is important to note that histone lactylation is driven by nuclear Lactyl-CoA, whose precursor is intracellular free lactate. The kinetics of lactate across cell membranes (via MCT1/MCT4) and nuclear membranes are complex. In the SjD inflammatory microenvironment, MCT1 and MCT4 expression may be altered, potentially influencing intracellular lactate concentrations and subsequent nuclear lactylation. Whether MCT upregulation leads to increased lactate efflux (lowering intracellular lactate) or increased uptake (raising intracellular lactate) in specific cell types requires further investigation (58).

### 6.2 Lactylation-driven fibrogenesis

Fibrosis represents the primary pathological basis for progressive loss of salivary gland function in SjD (4). Beyond SjD, the mechanisms by which lactylation participates in fibroblast activation and extracellular matrix (ECM) deposition have been extensively reported. In pulmonary fibrosis models, TGF-β1 stimulation induces glycolytic reprogramming and lactate production in lung fibroblasts, accompanied by elevated H3K18la at COL1A1, COL3A1, and ACTA2 promoters; p300/CBP inhibition or LDHA knockdown attenuates fibroblast activation (59). In hepatic stellate cell activation, genome-wide lactylation profiles undergo rearrangement, with pro-fibrotic gene loci showing H3K18la enrichment (60). In cardiac fibrosis models, macrophage-derived lactate is taken up by fibroblasts through paracrine mechanisms, inducing histone lactylation and α-SMA expression (28). Although direct evidence in SjD salivary gland fibroblasts is lacking, this conserved mechanism across fibrotic diseases suggests a potentially similar role for lactylation in SjD-associated fibrogenesis. The developmental lineage of fibroblasts may influence their response to lactate: salivary gland fibroblasts derive from neural crest and mesenchyme, whereas liver and heart fibroblasts are mesodermal in origin (61). Despite these differences, the core TGF-β/Smad and glycolytic pathways are conserved, supporting the plausibility of lactylation-driven fibrosis in SjD.

### 6.3 Lactylation and epithelial-mesenchymal transition

Epithelial-mesenchymal transition (EMT) refers to the process by which epithelial cells lose polarity and epithelial markers (E-cadherin), acquiring mesenchymal phenotypes (vimentin, α-SMA) and migratory capacity (4). In SjD salivary glands, SGECs undergo EMT under sustained TGF-β, IL-6, and TNF-α stimulation, considered an important precursor to acinar loss, ductal hyperplasia, and ECM deposition. EMT-derived myofibroblasts secrete ECM components, directly participating in fibrotic remodeling, while transformed SGECs show decreased aquaporin-5 expression and impaired secretory function (62).

In multiple disease models, histone lactylation drives EMT-related gene transcription. In breast and lung cancer cells, lactate promotes H3K18la enrichment at SNAI1 and ZEB1 promoter regions (63). In liver fibrosis, TGF-β-induced glycolytic reprogramming upregulates TWIST1 promoter lactylation (64). Hypoxic signals in the SjD microenvironment strengthen this coupling: HIF-1α directly induces EMT transcription factor expression while driving glycolytic enzyme transcription, increasing lactate production. Lactate then establishes open chromatin states at EMT gene loci through histone lactylation, forming a HIF-1α-glycolysis-lactylation-EMT positive feed-forward loop (65). Although this loop has been validated in tumors and other fibrotic diseases, whether it operates in SjD SGECs awaits experimental confirmation. Importantly, histone lactylation may also contribute to acinar cell atrophy and destruction, directly linking this epigenetic modification to the clinical symptom of hyposalivation (66).

### 6.4 Lactylation-mediated immune cell regulation in SjD

As detailed in Section 4, lactylation regulates macrophage, T cell, and B cell function. In SjD, salivary gland-infiltrating macrophages are predominantly M1-type (27); Th17/Treg imbalance is a recognized feature (23); and B cell hyperactivation drives autoantibody production (50). While direct evidence for lactylation’s role in these processes in SjD is lacking, the conserved mechanisms across other diseases strongly suggest lactylation as a key regulator of the immune dysregulation central to SjD pathogenesis.

### 6.5 Trained immunity and lactylation

Trained immunity is the long-term functional reprogramming of innate immune cells following initial inflammatory stimuli (67). Primary SjD exhibits features consistent with trained immunity, including sustained monocyte and macrophage hyperresponsiveness, elevated baseline cytokine production, and glycolytic reprogramming (68). The epigenetic basis of trained immunity involves stable alterations in histone modifications at inflammatory gene promoters (69). Histone lactylation may play an underappreciated role in establishing and maintaining trained immunity phenotypes (70). In SjD, pDCs and monocytes chronically activated by endogenous nucleic acids and type I IFN may establish a lactylation-dependent trained state capable of maintaining inflammatory responses even in the absence of persistent triggering stimuli (71). This trained immunity hypothesis provides a mechanistic framework for understanding disease chronicity and flares in SjD.

## 7 Therapeutic implications

The recognition of histone lactylation as a potential driver of Sjögren’s disease (SjD) pathology raises the question of whether existing or emerging therapies for SjD may exert beneficial effects, at least in part, through modulating the lactate–lactylation axis. Conversely, it also suggests that directly targeting the lactylation machinery could represent a novel therapeutic strategy. It is important to emphasize that no drug specifically designed to inhibit histone lactylation has yet entered clinical trials for SjD. The following discussion therefore serves two purposes: (1) to examine how current and emerging SjD therapeutics may intersect with the metabolic–epigenetic pathways discussed in this review, and (2) to highlight preclinical strategies targeting lactylation that may inform future drug development for SjD. A summary of current and emerging therapeutic strategies for Sjögren’s disease (SjD) and their potential intersection with the lactate–lactylation axis is provided in **Table 1**.

**Table 1:** Therapeutic strategies for Sjögren’s disease and their potential intersection with the lactate-lactylation axis.

Category	Agent/target	Mechanism of action	Clinical evidence in SjD/autoimmunity	Potential intersection with lactate-lactylation axis	Key challenges/considerations
B-Cell-Targeted Therapies	Ianalumab (VAY736)	Dual B-cell depletion (ADCC) and BAFF-R inhibition	Phase III – NEPTUNUS-1 and -2 met primary endpoints; first phase III success in SjD	Reduces B-cell burden in germinal centers; may lower local lactate production and subsequent lactylation at immunoglobulin loci	Long-term safety; impact on humoral immunity; biomarkers for patient selection
B-Cell-Targeted Therapies	Telitacicept (RC18)	TACI-Fc fusion protein; blocks BAFF and APRIL	Phase III – Significant ESSDAI reduction at week 24 vs placebo	Similar to ianalumab; APRIL blockade may further reduce plasma cell survival and local inflammation	Long-term immunogenicity; optimal dosing regimen
B-Cell-Targeted Therapies	Rituximab	Anti-CD20 monoclonal antibody; B-cell depletion	Phase III (mixed) – Failed primary endpoints but widely used off-label	B-cell depletion may reduce germinal center glycolysis; inconsistent clinical effects suggest indirect impact on lactylation	Discrepancy between trial results and real-world use; need for biomarker-guided patient selection
B-Cell-Targeted Therapies	Belimumab	Anti-BAFF monoclonal antibody	Phase II – 60% response at 6 months; approved for SLE	Reduces BAFF-driven B-cell survival and associated metabolic activation	Less robust evidence in SjD vs SLE; combination strategies under investigation
Co-Stimulatory Pathway Inhibitors	Dazodalibep (DAZ)	CD40 ligand antagonist (non-antibody fusion protein)	Phase II – Met endpoints in both systemic and symptomatic SjD populations	Reduces T-B cell crosstalk and downstream T-cell glycolytic activation	Novel mechanism; larger phase III trials needed
Co-Stimulatory Pathway Inhibitors	Iscalimab (CFZ533)	Anti-CD40 monoclonal antibody	Phase IIb – Significant dose-response relationship for ESSDAI	Similar to dazodalibep; CD40 blockade may reduce antigen presentation-driven T-cell metabolic reprogramming	Dose optimization; long-term safety data pending
Intracellular Signaling Inhibitors	Tofacitinib	JAK1/JAK3 inhibitor	Phase Ib/IIa – Initiated; ex vivo data show normalized JAK-STAT signaling	Directly interrupts IFN-JAK-STAT-HIF-1α-glycolysis axis; may reduce lactate substrate for histone lactylation	Infection risk; lipid profile effects; off-target JAK inhibition
Intracellular Signaling Inhibitors	Baricitinib	JAK1/JAK2 inhibitor	Phase II – Significant ESSDAI improvement reported	Similar to tofacitinib; may also modulate macrophage metabolism	Long-term safety data needed in SjD; efficacy vs JAK1-selective agents
Intracellular Signaling Inhibitors	Remibrutinib (LOU064)	BTK inhibitor (covalent, selective)	Phase II – Significant ESSDAI improvement; ~50% reduction in CXCL13	Modulates B-cell and Fc receptor signaling; may reduce inflammatory milieu that sustains glycolysis	Hepatotoxicity monitoring; drug-drug interactions
Intracellular Signaling Inhibitors	Deucravacitinib	TYK2 inhibitor (allosteric)	Phase II – Being explored in SjD	Modulates type I IFN and IL-12/23 pathways; may indirectly affect glycolytic gene expression	Specificity for TYK2 vs other JAK family members; efficacy in SjD not yet established
Immunometabolic Modulators	Iguratimod (IGU)	Anti-inflammatory agent; modulates metabolic pathways	Phase II/III – Superior to HCQ for STAR (39.5% vs 15.0%, P = 0.015) and IgG reduction	Shown to modulate immune cell metabolism; direct effects on histone lactylation unknown	Mechanistic studies on lactylation needed; manageable safety profile
Immunometabolic Modulators	Hydroxychloroquine (HCQ)	Lysosomotropic agent; TLR signaling inhibitor	Clinical trials (limited) – Modest efficacy; widely used off-label	Indirect effects on metabolic signaling via lysosomal function; unclear relevance to lactylation	Limited efficacy; retinal toxicity with long-term use
Preclinical Lactylation-Targeting Strategies (Autoimmune Models)	Paederoside	Natural compound; inhibits SMAD4-MCT1 axis → reduces lactate transport and H3K14la	Preclinical (CIA model) – Alleviates arthritis; shifts M1→M2 macrophage polarization	Directly suppresses histone lactylation (H3K14la) in senescent macrophages; validates SMAD4-MCT1-lactylation axis as therapeutic target	Not yet tested in SjD; requires validation in salivary gland microenvironments
Preclinical Lactylation-Targeting Strategies (Autoimmune Models)	Baicalin	Natural flavonoid; inhibits HIF-1α-driven glycolysis	Preclinical (autoimmune uveitis) – Reduces lactate, H3K18la, and NET formation	Counteracts HIF-1α-glycolysis-lactylation axis; potential applicability to macrophage/neutrophil-mediated tissue damage	Not tested in SjD; bioavailability in salivary gland unknown
Preclinical Lactylation-Targeting Strategies (Autoimmune Models)	FX-11	LDHA inhibitor; reduces lactate production	Preclinical (CIA model) – Reduces H3K9la and NFATc2; inhibits RA synoviocyte migration; additive with methotrexate	Directly reduces lactate substrate for histone lactylation; validated in autoimmune arthritis context	Not tested in SjD; systemic LDHA inhibition may cause muscle toxicity
Preclinical Lactylation-Targeting Strategies (Autoimmune Models)	AZD-3965	MCT1 inhibitor; blocks lactate transport	Preclinical (CIA model) – Similar efficacy to FX-11; reduces H3K9la	Disrupts lactate uptake/efflux; affects histone lactylation in paracrine signaling contexts	Phase I/II in tumors; not tested in SjD; target cell specificity unclear

### 7.1 B-cell-targeted therapies

B-cell dysregulation is a central feature of SjD, and B-cell-directed therapies represent the most advanced class of investigational agents for this disease.

Ianalumab (VAY736), a novel human IgG1 monoclonal antibody that achieves dual B-cell depletion via antibody-dependent cell-mediated cytotoxicity and BAFF-receptor inhibition, has demonstrated promising efficacy in clinical trials. A phase IIb dose-finding trial demonstrated that subcutaneous ianalumab significantly reduced disease activity and improved patient-reported outcomes in primary SjD (72). These outcomes represent a potential breakthrough toward the first disease-modifying therapy specifically approved for this condition. From a metabolic–epigenetic perspective, BAFF signaling promotes B-cell survival and activation, which is accompanied by enhanced glycolytic metabolism and lactate production. Whether ianalumab-mediated B-cell depletion reduces local lactate accumulation and subsequent histone lactylation in salivary gland germinal centers remains an open question.

Telitacicept (RC18), a recombinant TACI-Fc fusion protein that blocks both BAFF and APRIL signaling, has also shown promising efficacy. A phase III confirmatory clinical study (UPSTREAM SjD) met its primary endpoint, with results presented as an oral report at the 2026 EULAR congress, demonstrating that telitacicept significantly reduced ESSDAI scores in patients with active primary SjD (73).

Rituximab, an anti-CD20 monoclonal antibody, has generated extensive but mixed evidence in SjD. While earlier phase III trials failed to meet primary endpoints (74, 75), rituximab remains widely used off-label for severe systemic manifestations. Belimumab, an anti-BAFF monoclonal antibody approved for systemic lupus erythematosus, has shown benefit in phase II trials of SjD patients, with 60% of patients responding at six months (76).

### 7.2 Co-stimulatory pathway inhibitors

The CD40/CD40L costimulatory pathway plays a critical role in T–B cell interactions and has emerged as a promising therapeutic target in SjD.

Dazodalibep (DAZ), a novel non-antibody fusion protein that acts as a CD40 ligand antagonist, blocks costimulatory signals between T cells, B cells, and antigen-presenting cells. In a phase II randomized, double-blinded, placebo-controlled trial, DAZ met its primary endpoints in two distinct SjD populations, demonstrating significant reductions in disease activity and substantial improvement in symptoms (77).

Iscalimab (CFZ533), a monoclonal antibody against CD40, was evaluated in the phase IIb TWINSS study. At week 24, iscalimab demonstrated improvements over placebo in both populations studied and showed important signals for improvement in salivary flow, with a generally well-tolerated safety profile (78).

The relevance of these co-stimulatory inhibitors to histone lactylation lies in their ability to reduce T-cell activation and subsequent glycolytic reprogramming. Activated T cells undergo a metabolic switch toward aerobic glycolysis, producing lactate that can drive histone lactylation in both autocrine and paracrine manners. By dampening T-cell activation, CD40L antagonists may indirectly reduce the local lactate burden and consequent lactylation in the salivary gland microenvironment.

### 7.3 Intracellular signaling inhibitors

JAK inhibitors have a strong mechanistic rationale in SjD, given the central role of the type I interferon–JAK–STAT pathway in disease pathogenesis. Tofacitinib, a JAK1/JAK3 inhibitor, has been evaluated in patients with primary SjD, demonstrating significant ESSDAI reduction at 6 months (P = 0.001) and suppression of T follicular helper and T peripheral helper cells (79).

Baricitinib, a JAK1/JAK2 inhibitor, has also shown promise in active primary SjD patients, with a pilot study demonstrating its effectiveness and safety (80). Given that JAK–STAT signaling drives HIF-1α-mediated glycolysis and lactate production, JAK inhibitors may exert dual benefits: suppressing inflammation directly while reducing the metabolic substrate for histone lactylation.

BTK inhibitors represent another class of intracellular signaling inhibitors with promise in SjD. Remibrutinib (LOU064), a potent, highly selective covalent BTK inhibitor, was evaluated in a phase II randomized, double-blind, placebo-controlled trial, demonstrating significant improvement in ESSDAI score and unstimulated salivary flow, along with a favorable safety profile (81). BTK is a key signaling node in B-cell and Fc receptor signaling, and its inhibition may modulate both B-cell function and the inflammatory milieu that drives lactylation.

TYK2 inhibitors such as deucravacitinib, which modulate type I interferon and IL-12/23 signaling, are also being explored in SjD.

### 7.4 Immunometabolic modulators

Several agents originally developed for other indications are being repurposed for SjD based on their immunomodulatory and metabolic effects.

Iguratimod (IGU), an anti-inflammatory and immunosuppressive agent approved for rheumatoid arthritis, has been evaluated in SjD. A multicenter randomized controlled trial demonstrated that IGU was superior to hydroxychloroquine for key endpoints including STAR response (39.5% vs 15.0%, P = 0.015) and ESSDAI response (21.1% vs 5.0%, P = 0.034), as well as superior IgG reduction (82). Notably, IGU has been shown to modulate metabolic pathways in immune cells, though its direct effects on histone lactylation remain to be investigated.

Hydroxychloroquine (HCQ), despite its limited efficacy in clinical trials (83), remains widely used off-label for SjD. HCQ is known to affect lysosomal function and may indirectly influence metabolic signaling, but its relevance to the lactate–lactylation axis is unclear and warrants further study.

### 7.5 Preclinical strategies targeting histone lactylation in autoimmune disease models

While no lactylation-targeting drug has entered SjD clinical trials, recent preclinical studies in other autoimmune diseases have provided proof-of-concept evidence that directly inhibiting histone lactylation is therapeutically feasible.

In rheumatoid arthritis (RA), a landmark study demonstrated that the natural compound paederoside alleviates arthritis by targeting SMAD4 to inhibit MCT1-mediated lactate transport, thereby suppressing histone lactylation (H3K14la) in senescent macrophages (84). This resulted in reduced pro-inflammatory cytokine expression, a shift toward M2 macrophage polarization, and attenuated joint inflammation in the collagen-induced arthritis model. The SMAD4–MCT1 axis was identified as a novel metabolic–epigenetic target for RA therapy.

In autoimmune uveitis, baicalin was shown to counteract HIF-1α-driven glycolysis, leading to reduced lactate production, decreased H3K18la levels, and suppression of neutrophil extracellular trap formation (85). This study established a mechanistic axis in which HIF-1α-driven glycolysis promotes lactate-dependent histone lactylation, and pharmacological inhibition of this pathway alleviates autoimmune inflammation.

In RA fibroblast-like synoviocytes, pharmacological inhibition of lactate production (via the LDHA inhibitor FX-11) or lactate transport (via the MCT1 inhibitor AZD-3965) reduced H3K9la levels and NFATc2 expression, thereby inhibiting cell migration and cytokine secretion. In the CIA mouse model, both FX-11 and AZD-3965 alleviated arthritis severity and, when combined with methotrexate, produced additive therapeutic effects (86).

These preclinical findings have direct implications for SjD. The salivary gland microenvironment in SjD shares key features with the inflamed synovium in RA and the ocular tissues in uveitis—namely, glycolytic immune cells, lactate accumulation, and histone lactylation-driven inflammatory gene expression. The SMAD4–MCT1–H3K14la axis identified in RA macrophages may have analogous counterparts in SjD-infiltrating macrophages and salivary gland epithelial cells. Similarly, the HIF-1α–glycolysis–H3K18la–NETosis pathway in uveitis raises the question of whether neutrophil or macrophage lactylation contributes to salivary gland tissue damage in SjD.

However, several critical differences must be acknowledged. RA is a destructive joint disease with a different tissue microenvironment and cellular composition compared to SjD salivary glands. The pharmacokinetics of compounds like paederoside, baicalin, FX-11, and AZD-3965 in salivary gland tissue have not been characterized. Moreover, the relative contribution of histone versus non-histone lactylation to SjD pathogenesis remains unknown. These considerations underscore the need for SjD-specific preclinical models to validate these therapeutic strategies before any clinical translation can be contemplated.

### 7.6 Summary and outlook

The therapeutic landscape of SjD is undergoing a remarkable transformation. After decades with no approved disease-modifying therapies, multiple phase II and III trials have now demonstrated meaningful efficacy, raising the possibility of the first targeted treatments for SjD in the near future.

From the perspective of histone lactylation biology, several emerging themes are worth highlighting:

B-cell-targeted therapies (ianalumab, telitacicept) and co-stimulatory inhibitors (dazodalibep, iscalimab) may indirectly reduce lactylation by dampening immune activation and the associated glycolytic metabolism.

JAK inhibitors (tofacitinib, baricitinib) may directly interrupt the IFN–JAK–STAT–HIF-1α–lactate axis that provides substrate for histone lactylation.

Immunometabolic modulators (iguratimod) offer a bridge between conventional immunosuppression and metabolic reprogramming, though their effects on lactylation require investigation.

Preclinical strategies directly targeting lactate production, transport, or histone lactylation (FX-11, AZD-3965, paederoside, baicalin) have shown efficacy in RA and uveitis models, providing a roadmap for future SjD research.

Critically, none of the lactylation-targeting compounds discussed above has been evaluated in SjD. The path from preclinical proof-of-concept to clinical application in SjD will require: (1) comprehensive lactylome profiling of SjD salivary gland tissues to identify disease-relevant lactylation sites and cell types; (2) validation of candidate compounds in SjD-relevant *in vitro* systems (e.g., primary SGECs, patient-derived fibroblasts, and infiltrating immune cells); (3) efficacy testing in SjD animal models such as the NOD/ShiLtJ mouse; and (4) careful consideration of safety, given that systemic inhibition of glycolysis or histone lactylation may have off-target effects on normal tissues.

Nevertheless, the convergence of advances in SjD clinical trials and the rapidly expanding understanding of lactylation biology creates an unprecedented opportunity. We propose that histone lactylation should be considered not only as a pathogenic mechanism but also as a potential biomarker for patient stratification and a therapeutic target in future SjD clinical trials.

## 8 Future directions and conclusions

### 8.1 Future research directions

Despite remarkable progress, several critical gaps must be filled to elucidate lactylation’s role in SjD. First, comprehensive lactylome profiling of SjD patient samples using quantitative mass spectrometry and ChIP-sequencing at single-base resolution is urgently needed to identify disease-specific lactylation patterns. Such analyses should be performed on purified cell populations from salivary gland biopsies, including SGECs, fibroblasts, macrophages, and T cells. Second, development of SjD-specific animal models incorporating lactylation reporters and conditional knockout/knock-in strategies for key enzymes would enable mechanistic studies. The NOD/ShiLtJ mouse model could be engineered to express H3K18la-specific FRET probes to visualize lactylation dynamics. Third, establishment of standardized methodologies for lactylation detection is essential. Development of site-specific antibodies validated through peptide competition and mass spectrometry would improve specificity. Additionally, single-cell lactylome analysis technologies will reveal heterogeneity in lactylation patterns within mixed cell populations.The absence of lactylation-targeting agents in SjD clinical trials, as discussed in Section 7, underscores the urgent need for comprehensive basic and translational research to validate histone lactylation as a viable therapeutic target in this disease.

### 8.2 Conclusions

Histone lactylation represents a transformative discovery bridging immunology, metabolism, and epigenetics. In Sjögren’s disease, the convergence of chronic inflammation, tissue hypoxia, and metabolic reprogramming creates favorable conditions for pathological histone lactylation in salivary gland-resident and infiltrating cells. Accumulating evidence supports a model in which elevated lactate production drives lactylation-dependent gene expression programs that promote immune dysregulation, epithelial-mesenchymal transition, and myofibroblast activation, ultimately leading to progressive glandular fibrosis and secretory dysfunction. While direct evidence in SjD is currently lacking, the conserved mechanisms observed across multiple inflammatory and fibrotic diseases provide a compelling framework. The therapeutic implications of this framework are twofold. At the clinical level, emerging SjD therapeutics—including B-cell-targeting agents (ianalumab, telitacicept), co-stimulatory inhibitors (dazodalibep, iscalimab), JAK inhibitors (tofacitinib, baricitinib), and immunometabolic modulators (iguratimod)—may exert beneficial effects, at least in part, by dampening immune activation and the associated glycolytic metabolism, thereby indirectly reducing lactate availability for histone lactylation. At the preclinical level, direct pharmacological targeting of the lactylation machinery (via LDHA inhibitors, MCT1 inhibitors, or natural compounds such as paederoside and baicalin) has shown promise in autoimmune disease models, providing a roadmap for future SjD research.The proposed metabolic-epigenetic vicious cycle provides a unifying model for understanding the transition from reversible inflammation to irreversible fibrosis in SjD. Intervention at any point in this cycle—whether through modulating immune cell activation to reduce lactate production, or through emerging preclinical strategies targeting the lactylation machinery—represents a rational therapeutic strategy. Critically, none of the direct lactylation-targeting approaches has yet been evaluated in SjD, and the path from preclinical proof-of-concept to clinical application will require rigorous validation in SjD-specific models. Nevertheless, the convergence of advances in SjD clinical trials and the rapidly expanding understanding of lactylation biology creates an unprecedented opportunity. We propose that histone lactylation should be considered not only as a pathogenic mechanism but also as a potential biomarker for patient stratification and a therapeutic target in future SjD clinical trials.
